# Diagnosis of Rhinocerebral Mucormycosis by Treatment of Cavernous Right Internal Carotid Artery Occlusion With Mechanical Thrombectomy: Special Case Presentation and Literature Review

**DOI:** 10.3389/fneur.2019.00264

**Published:** 2019-03-26

**Authors:** Samir Kashyap, Jacob Bernstein, Hammad Ghanchi, Ira Bowen, Vladimir Cortez

**Affiliations:** ^1^Department of Neurosurgery, Riverside University Health System, Moreno Valley, CA, United States; ^2^Department of Neurosurgery, Arrowhead Regional Medical Center, Colton, CA, United States; ^3^Redlands Community Hospital, Redlands, CA, United States

**Keywords:** mucormycosis, thrombectomy, antifungal, rhinocerebral mucomycosis, posaconazole

## Abstract

**Background:** Mucormycosis is a rapidly progressive, angioinvasive fungal infection that has a predilection for the paranasal sinuses and adjacent mucosa. Rhinocerebral mucormycosis (RCM) is the most common form and is known to invade the skull base and its associated blood vessels—leading to mycotic aneurysms, ischemic infarcts, and intracerebral hemorrhage. There are documented cases of mechanical thrombectomy in ischemic stroke due to RCM, however, there are no known cases that were diagnosed primarily by histological and pathological analysis of the embolus. We present a case of treatment of large vessel occlusion that led to the diagnosis and treatment of RCM.

**Case Presentation:** A 21 year-old male inmate with history of type 1 diabetes presented with generalized weakness, abdominal pain, right eye blindness, and ophthalmoplegia after an assault in prison. He underwent treatment for diabetic ketoacidosis, but subsequently developed left hemiplegia and was found to have complete occlusion of his right internal carotid artery. He underwent successful mechanical thrombectomy and pathological analysis of the embolus revealed a diagnosis of mucormycosis. He completed a course of amphotericin B, micafungin, and posaconazole. With the aid of acute rehabilitation he achieved a modified Rankin score of 2.

**Discussion:** We review the pathogenesis, diagnosis, and treatment of RCM. A comprehensive multidisciplinary approach is critical in the management of this often-fatal disease. Early diagnosis and treatment are essential in RCM as delaying treatment by more than 6 days significantly increases mortality. Treatment includes surgical debridement and intravenous antifungal therapy (amphotericin B + micafungin or caspofungin) for a minimum of 6–8 weeks.

## Background

Mucormycosis is a rare opportunistic fungal infection that is rapidly progressive and often fatal despite the availability and utilization of the most potent antifungal agents (1–4). The disease can also be referred as zygomycosis or phycomycosis. Patients at highest risk for infection include patients with weakened immune systems, poorly controlled diabetics, kidney failure, organ transplants, chemotherapy patients, and those with severe immunocompromise (1, 2, 5, 6). Clinical manifestations of mucormycosis infection often include cutaneous, pulmonary, rhinocerebral, gastrointestinal, and disseminated sepsis. Rhinocerebral mucormycosis (RCM) is the most common form (3). The disease's preference for paranasal sinuses and adjacent mucosa is not well-understood; however, it is thought that inhalation of spores results in primary infection and colonization within the nasal mucosa (6). After colonization, involvement of the adjacent paranasal sinuses and skull often occurs via contiguous spread of the disease (6). This can result in devastating neurological sequences due to its preference for cerebral vasculature. Cases of mycotic aneurysm, infarction, and hemorrhage associated with RCM are well-documented. The authors present a rare case of mucormycosis infection with large vessel occlusion (LVO) of the right internal carotid artery (RICA) with the patient undergoing successful mechanical thrombectomy for recannulation of the RICA. The clot was sent for cultures and pathology with confirmation of fungi consistent with Mucormycosis. The patient was subsequently treated with amphotericin B, micafungin, and posaconazole.

## Case Presentation

A 21-year-old male inmate presented to our facility with generalized weakness, abdominal pain, nausea, and right eye pain with associated inflammation and blindness. The patient reported that, 3 days prior to arrival, he was involved in an altercation where he was struck in the face and had feces smeared over the right side of his face. Past medical history was significant for type 1 diabetes mellitus and methamphetamine abuse.

### Physical Exam

On admission, the patient was in acute distress due to lack of vision in his right eye. The patient's clinical examination was consistent with orbital apex syndrome with injury and inflammation in the cavernous sinus. The right eye had a fixed, non-reactive pupil and exhibited ophthalmoplegia, scleral injection, periorbital edema, and erythema. Remaining physical and neurological examination were normal.

### Pertinent Laboratory Values

On admission, his glucose was 437 mg/dL, Hb A1c was 14.5%, ß-hydroxybutryate/acetoacetate ratio was 9.00, HCO_3_: 5 meQ/L, WBC 21.7, Urinalysis: 4+ ketones, 4+ glucose, and urine drug screen (UDS) was negative.

### Clinical Course

The patient was admitted to the intensive care unit (ICU) under our institution's diabetic ketoacidosis (DKA) protocol where he remained for 48 h. Ophthalmology was consulted for his right orbital cellulitis and ophthalmoplegia and Oral and Maxillofacial Surgery (OMFS) was consulted for reducing his nasal fracture. Soft tissue cultures were taken of the region. No interventions were recommended at the time by either service except for intravenous antibiotics. There was no suspicion for fungal infection on their respective evaluations. After resolution of DKA, he was transferred to the general floor. During this time, he experienced no changes in neurological exam or in his ocular exam.

**Figure 1:**
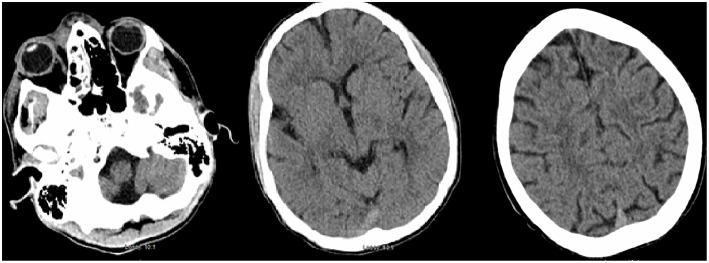
Initial CT head demonstrating periorbital edema and inflammation in the ethmoid and maxillary sinuses. There is no evidence of acute intracranial abnormality.

**Figure 2:**
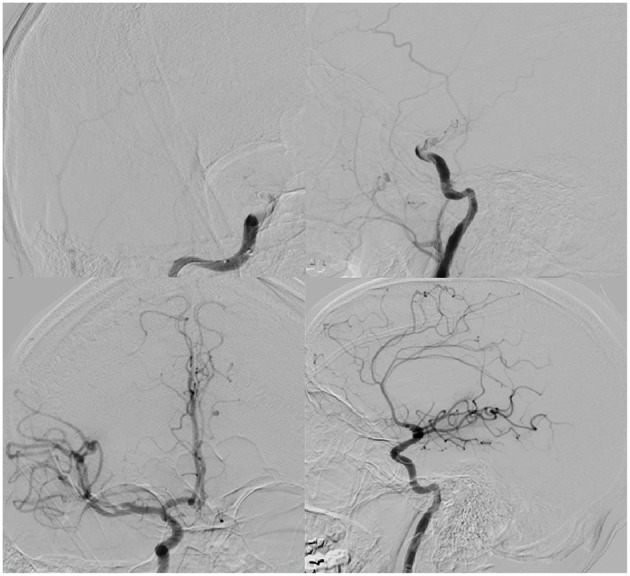
**(Top)**: AP (left) and lateral (right) digital subtraction angiogram illustrating right ICA terminus occlusion on common carotid injection. **(Bottom):** AP (left), lateral (right) demonstrating TICI III recanalization after thrombectomy.

Less than 24 h after transfer, the patient developed sudden-onset left facial droop, gaze preference to the right and left-sided hemiplegia. Immediate CT of the head revealed no obvious acute abnormalities (Figure 1); however, CTA head demonstrated occlusion of the cavernous segment of the RICA. The patient was immediately evaluated by the on-call vascular neurologist where he was diagnosed with an acute ischemic stroke with LVO of RICA and right MCA syndrome. Subsequently, the patient was administered IV-tPA per our institution's stroke protocol. The patient did not improve in his symptoms after completion of IV-tPA. Neurointerventional surgery was consulted for possible mechanical thrombectomy and the patient was immediately taken for a cerebral angiogram, confirming complete occlusion of the RICA. Utilizing standard thrombectomy techniques, a TICI III (Thrombolysis in Cerebral Infarction) revascularization was achieved (Figure 2) using a retrievable stent system. Direct evaluation of the embolus did not seem consistent with a standard thrombus as it had a fibrous appearance with minimal blood product. Given this finding, the specimen was sent for pathology and culture.

**Figure 3:**
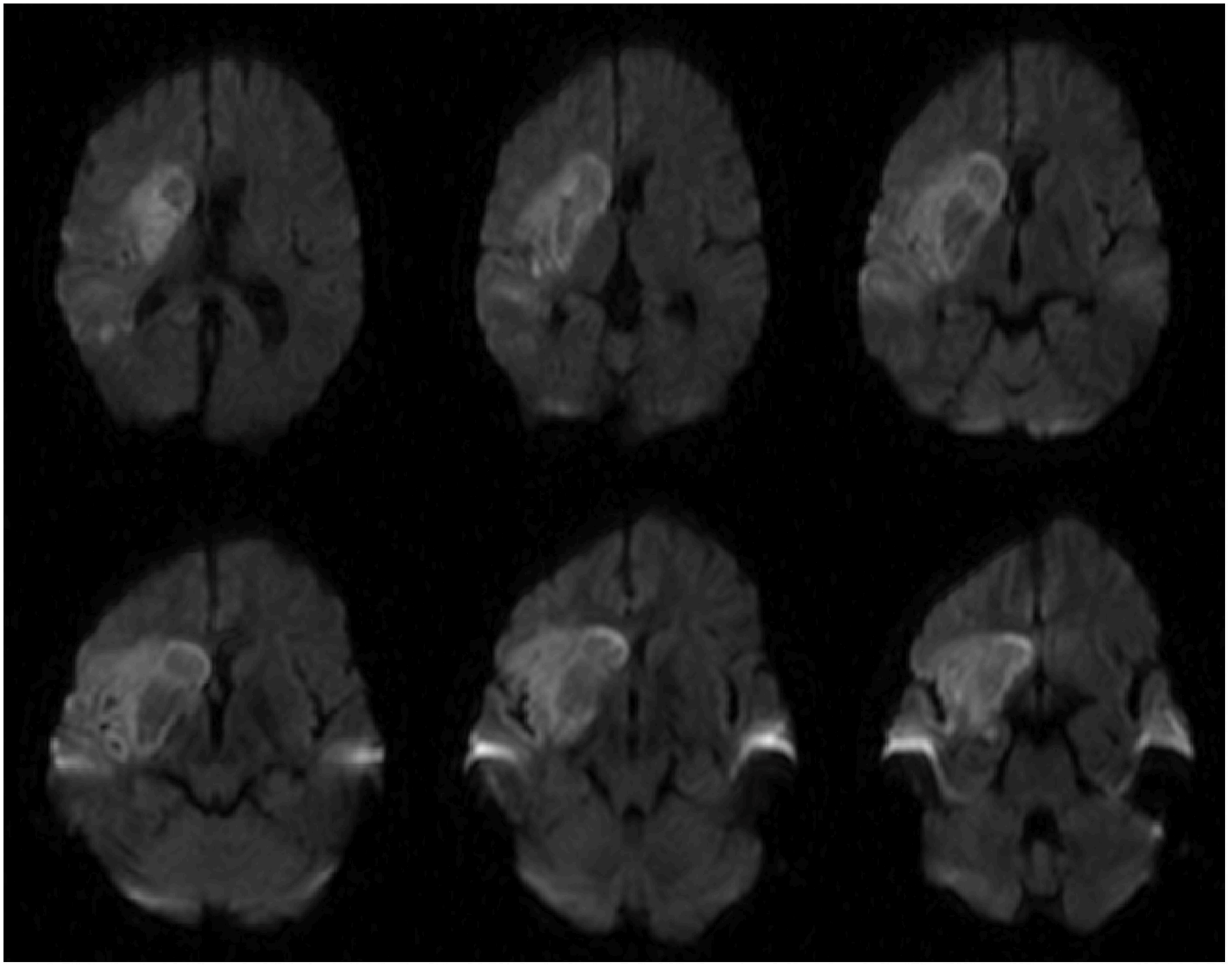
Diffusion weighted imaging (DWI) sequences demonstrating diffusion restriction in the basal ganglia that is characteristically seen in rhinocerebral mucormycosis.

**Figure 4:**
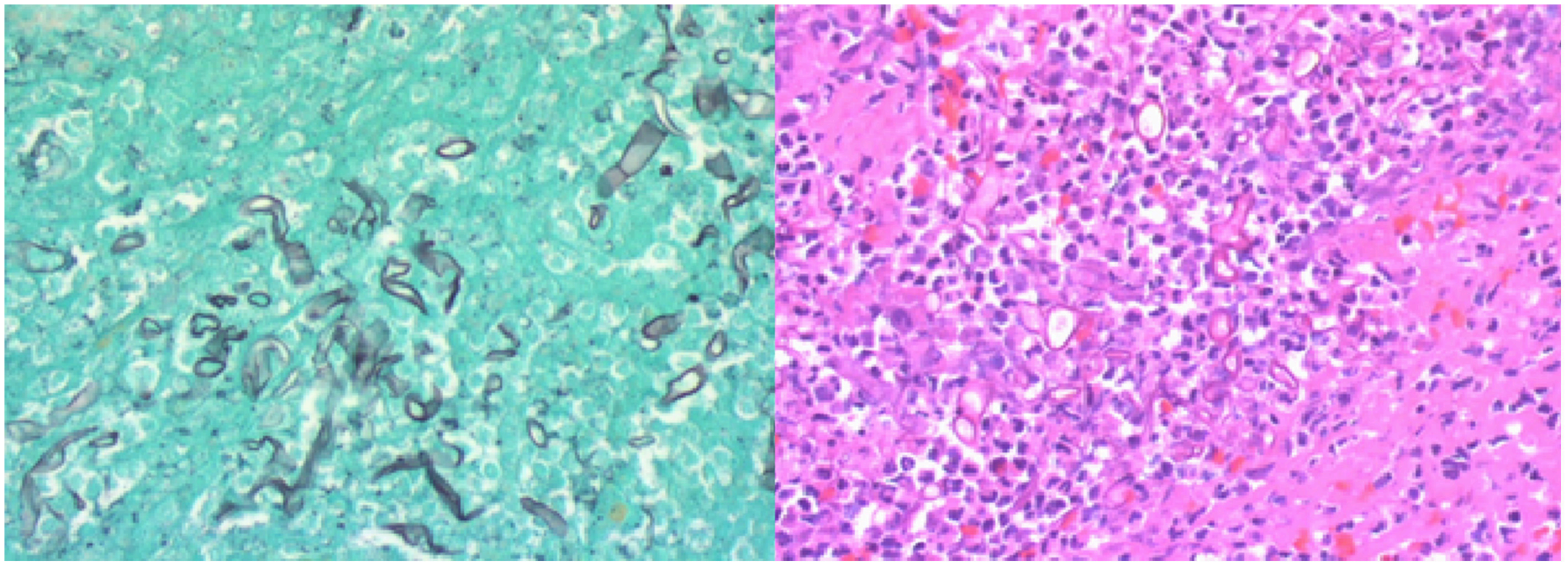
**(Left)** Gomori methenamine silver (GMS) stain highlighting fungal wall and the pathognomonic non-septate branching hyphae. **(Right)** High-power H&E stain demonstrating hyphae and inflammatory cells with limited blood product. Photo Credit: Sin Nguyen, MD.

The patient recovered in the ICU after the procedure. Post-procedural MRI demonstrated diffusion restriction over the right basal ganglia, middle and inferior frontal lobes, and anterior temporal lobe (Figure 3). Final pathology and culture were positive for fungal hyphae consistent with RCM (Figure 4). Additional blood cultures were positive for multi-drug resistant *Klebsiella pneumoniae*. Once the diagnosis was confirmed, the patient was started on micafungin, amphotericin B, and meropenem. He then underwent urgent ethmoidectomy and maxillary antrostomy with OMFS followed by right orbital exenteration with ophthalmology. He completed an 8-week course of IV micafungin, amphotericin B, and meropenem per infectious disease recommendations. The patient was transitioned to oral posaconazole for an additional 3 months. The patient recovered to a Modified Rankin Scale of 2 at 90 days with the aid of acute rehabilitation.

## Discussion

Mucormycosis is a rare, angioinvasive opportunistic fungal pathogen affecting lung, sinuses, and brain. It is often seen in immunocompromised and poorly controlled diabetics as well as IV drug addicts, of which RCM is the most common form (7, 8). There is a higher incidence in acute leukemia (6). The most common presentation are headaches, fever, facial and orbital swelling, and orbital apex syndrome (9, 10). Nearly all these presenting signs were seen in our case.

### Pathogenesis

Mucormycosis is inherently found in soil and decaying plants whose spores can be easily inhaled, making the nasal passages as the most common primary site of inoculation (11). The pathogen thrives as a scavenger of free iron and those individuals with elevated free serum iron levels appear to be at higher risk (5, 11). DKA significantly increases the risk of developing RCM due to the increased dissociation of free iron and the dysfunction of macrophages seen in acidic conditions, allowing the infection to propagate via its inherent ketone-reductase system (5, 12). The glucose-regulated protein 78 (GRP78) has been implicated in this pathogenesis and is shown in diabetic mice to be overexpressed in the progression of this disease (13). The primary mode of pathogenesis is hematogenous spread due to its ability to adhere to and invade the endothelium of neighboring blood vessels, allowing it to invade any organ system. A case of brain, lung, bone, and heart involvement as well an isolated case of renal involvement has also been reported (14). Thrombotic complications involving the cavernous sinus, ICA, ophthalmic, and retinal arteries are well-documented in the literature and significantly increase the morbidity associated with RCM (15, 16).

### Diagnosis

Cerebral infection and infarction, whether bacterial or fungal, are best diagnosed using magnetic resonance imaging (MRI) (17). The imaging findings characteristic of infection include rim enhancement on T1-post contrast imaging, central hyperintensity on T2 sequences, and diffusion restriction (18). Diffusion restriction is characteristically seen in the basal ganglia as mucormycosis has demonstrated an affinity for this location (19, 20). This finding was present in our patient (Figure 3), however it is more likely that this finding is due to infarct from his RICA occlusion. Contrasted imaging was not obtained due to the low suspicion of intracranial infection prior to thrombectomy so it is inconclusive whether this finding was due to infection or infarct.

A high index of suspicion of mucormycosis is necessary as an autopsy series demonstrated almost 50% of cases were diagnosed post-mortem (5). The most definitive diagnosis comes from culture and histological analysis (12). Traditional H&E staining is typically low yield for identification of *Mucorales*. Thus, utilizing Gomori methenamine silver (GMS) significantly increases the yield of diagnosis by highlighting the fungal wall (12). Broad, non-septate hyphae with right angle branching is the pathognomonic morphology seen on histological analysis (5, 12, 17, 20). Our case of RCM is particularly unique due to the fact that standard mechanical thrombectomy techniques provided the definitive diagnosis.

### Treatment/Prognosis

The prognosis of this disease is often fatal with mortality rates ranging from 33 to 80% (21). Early treatment and diagnosis is essential as Sipsas et al. demonstrated that delaying treatment by more than 6 days doubled the mortality rate (22, 23). The biggest risk factor for mortality is predictably those with disseminated infection (12, 21, 24). Aggressive surgical debridement is necessary in those patients with an identifiable lesion who are well enough to undergo an operation. This is particularly important in RCM to halt intracranial progression, improve outcomes, and response to systemic antifungal therapy (2–5, 21, 22, 25).

A comprehensive, multidisciplinary approach is necessary in these patients, of which our case is a prime example. Our patient underwent several medical and surgical interventions by multiple specialties. In addition to medical optimization of DKA and superimposed bacterial infections, neurointerventional surgery performed the thrombectomy which limited the size of potential infarct and provided the definitive diagnosis that was not made on initial workup. Ophthalmology and OMFS performed extensive surgical debridement of the affected orbit and paranasal sinuses after diagnosis to contain the infection.

First-line medical therapy is amphotericin B (5 mg/kg/day) and may include an additional IV antifungal agent such as caspofungin for 6–8 weeks (2, 4, 5, 19, 22, 26). Oral antifungals are often prescribed after the IV regimen is completed. Newer antifungals such as posaconazole and isavuconazole have shown promise as a salvage therapy (22, 23). This is especially important given the systemic adverse effects seen with amphotericin B (5, 23). In a review of 96 case reports, Vehreschild et al reported that posaconazole was able to achieve a complete response to in 64.6% of cases (27). This antifungal was used in our case after completion of amphotericin B and micafungin therapy and our patient has no evidence of disease 6 months after completion of antifungal therapy.

There are many documented cases in the literature of RCM invading the skull base and neighboring vasculature leading to ophthalmic, retinal, and ICA occlusion (1, 3, 6, 28). The unique nature of our case lies in the fact that the diagnosis of RCM was made via mechanical thrombectomy and histological analysis of the embolus. Due to the low suspicion of fungal infection at the time of initial presentation, the patient could have had an alternative and unfavorable clinical outcome.

## Conclusion

Mucormycosis is a rapidly progressive, angioinvasive disease most commonly seen in the brain and paranasal sinuses. Cerebral complications such as abscess and infarction due to the angioinvasive nature of this disease are well-documented. Early diagnosis, surgical debridement (when indicated), and antifungal therapy are essential in optimizing outcomes.

## Author Contributions

SK and VC contributed the conception and vision of the paper. SK, JB, HG, and IB contributed to drafting the paper, revising it critically, and literature review. All authors contributed to manuscript revision, read and approved the submitted version.

### Conflict of Interest Statement

The authors declare that the research was conducted in the absence of any commercial or financial relationships that could be construed as a potential conflict of interest.

## References

[1] Simmons JH Zeitler PS Fenton LZ Abzug MJ Fiallo-Scharer RV Klingensmith GJ. Rhinocerebral mucormycosis complicated by internal carotid artery thrombosis in a pediatric patient with type 1 diabetes mellitus: a case report and review of the literature. Pediatr Diabetes. (2005) 6:234–8. 10.1111/j.1399-543X.2005.00118.x 16390393

[2] Narayanan S Panarkandy G Subramaniam G Radhakrishnan C Thulaseedharan NK Manikath N The “black evil” affecting patients with diabetes: a case of rhino orbito cerebral mucormycosis causing Garcin syndrome. Infect Drug Resist. (2017) 10:103–8. 10.2147/IDR.S130926 28405168 PMC5378458

[3] Bae MS Kim EJ Lee KM Choi WS. Rapidly progressive rhino-orbito-cerebral mucormycosis complicated with unilateral internal carotid artery occlusion: a case report. Neurointervention. (2012) 7:45–9. 10.5469/neuroint.2012.7.1.45 22454785 PMC3299950

[4] Alvernia JE Patel RN Cai DZ Dang N Anderson DW Melgar M. A successful combined endovascular and surgical treatment of a cranial base mucormycosis with an associated internal carotid artery pseudoaneurysm. Neurosurgery. (2009) 65:733–40; discussion 740. 10.1227/01.NEU.0000351773.74034.5E 19834379

[5] Spellberg B Edwards J Ibrahim A. Novel perspectives on mucormycosis: pathophysiology, presentation, and management. Clin Microbiol Rev. (2005) 18:556–69. 10.1128/CMR.18.3.556-569.2005 16020690 PMC1195964

[6] Abela L Toelle SP Hackenberg A Scheer I Güngör T Plecko B Fatal outcome of rhino-orbital-cerebral mucormycosis due to bilateral internal carotid occlusion in a child after hematopoietic stem cell transplantation. Neuropediatrics. (2013) 44:1149–50. 10.1055/s-0033-1337839 24067555

[7] Delbrouck C Jacobs F Fernandez Aguilar S Devroede B Choufani G Hassid S. Carotid artery occlusion due to fulminant rhinocerebral mucormycosis. Acta Otorhinolaryngol Belg. (2004) 58:135–40. 15515658

[8] Borchard NA Nayak JV. Orbital apex syndrome. N Engl J Med. (2018) 378:e23. 10.1056/NEJMicm1703770 29694826

[9] Rumboldt Z Castillo M. Indolent intracranial mucormycosis: case report. Am J Neuroradiol. (2002) 23:932–4. 12063219 PMC7976923

[10] Raab P Sedlacek L Buchholz S Stolle S Lanfermann H. Imaging patterns of rhino-orbital-cerebral mucormycosis in immunocompromised patients : when to suspect complicated mucormycosis. Clin Neuroradiol. (2017) 27:469–75. 10.1007/s00062-017-0629-1 29026931

[11] Mititelu R Bourassa-Blanchette S Sharma K Roth V. Angioinvasive mucormycosis and paradoxical stroke: a case report. JMM Case Rep. (2016) 3:e005048. 10.1099/jmmcr.0.005048 28348771 PMC5330239

[12] Guarner J Brandt ME. Histopathologic diagnosis of fungal infections in the 21st century. Clin Microbiol Rev. (2011) 24:247–80. 10.1128/CMR.00053-10 21482725 PMC3122495

[13] Liu M Spellberg B Phan QT Fu Y Fu Y Lee AS. The endothelial cell receptor GRP78 is required for mucormycosis pathogenesis in diabetic mice. J Clin Invest. (2010) 120:1914–24. 10.1172/JCI42164 20484814 PMC2877958

[14] Lin CY Lee SC Lin CC Chan SC Lee CT. Isolated fatal renal mucormycosis in a patient with chronic obstructive pulmonary disease and tuberculosis. Int J Clin Pract. (2003) 57:916–8. 14712898

[15] Song YM Shin SY. Bilateral ophthalmic artery occlusion in rhino-orbito-cerebral mucormycosis. Korean J Ophthalmol. (2008) 22:66–9. 10.3341/kjo.2008.22.1.66 18323710 PMC2629957

[16] Frank GS Smith JM Davies BW Mirsky DM Hink EM Durairaj VD. Ophthalmic manifestations and outcomes after cavernous sinus thrombosis in children. J AAPOS. (2015) 19:358–62. 10.1016/j.jaapos.2015.06.001 26239205

[17] Gaviani P Schwartz RB Hedley-Whyte ET Ligon KL Robicsek A Schaefer P. Diffusion-weighted imaging of fungal cerebral infection. Am J Neuroradiol. (2005) 26:1115–21. 15891169 PMC8158608

[18] Terk MR Underwood DJ Zee CS Colletti PM. MR imaging in rhinocerebral and intracranial mucormycosis with CT and pathologic correlation. Magn Reson Imaging. (1992) 10:81–7. 10.1016/0730-725X(92)90376-B 1545686

[19] Hazama A Galgano M Fullmer J Hall W Chin L. Affinity of mucormycosis for basal ganglia in intravenous drug users: case illustration and review of literature. World Neurosurg. (2017) 98:872.e1–3. 10.1016/j.wneu.2016.11.130 27923750

[20] Weprin BE Hall WA Goodman J Adams GL. Long-term survival in rhinocerebral mucormycosis. Case report. J Neurosurg. (1998) 88:570–5. 10.3171/jns.1998.88.3.0570 9488314

[21] Hong H-L Lee Y-M Kim T Lee J-Y Chung Y-S Kim M-N. Risk factors for mortality in patients with invasive mucormycosis. Infect Chemother. (2013) 45:292–8. 10.3947/ic.2013.45.3.292 24396630 PMC3848522

[22] Sipsas NV Gamaletsou MN Anastasopoulou A Kontoyiannis DP. Therapy of Mucormycosis. J Fungi. (2018) 4:90. 10.3390/jof4030090 30065232 PMC6162664

[23] Shafiq M Ali Z Ukani R Brewer J. Isavuconazole: a promising salvage therapy for invasive mucormycosis. Cureus. (2018) 10:e2547. 10.7759/cureus.2547 29963341 PMC6021185

[24] Jayalakshmi SS Reddy RG Borgohain R Subramanyam C Panigrahi M Sundaram C. Predictors of mortality in rhinocerebral mycosis. Neurol India. (2007) 55:292–7. 10.4103/0028-3886.35692 17921660

[25] Luo QL Orcutt JC Seifter LS. Orbital mucormycosis with retinal and ciliary artery occlusions. Br J Ophthalmol. (1989) 73:680–3. 10.1136/bjo.73.8.680 2765451 PMC1041846

[26] Kazak E Aslan E Akalin H Saraydaroglu O Hakyemez B Erişen L. A mucormycosis case treated with a combination of caspofungin and amphotericin B. J Mycol Med. (2013) 23:179–84. 10.1016/j.mycmed.2013.06.003 23856448

[27] Vehreschild JJ Birtel A Vehreschild MJGT Liss B Farowski F Kochanek M. Mucormycosis treated with posaconazole: review of 96 case reports. Crit Rev Microbiol. (2013) 39:310–24. 10.3109/1040841X.2012.711741 22917084

[28] Patil A Mohanty HS Kumar S Nandikoor S Meganathan P. Angioinvasive rhinocerebral mucormycosis with complete unilateral thrombosis of internal carotid artery—case report and review of literature. BJR Case Rep. (2016) 2:20150448. 10.1259/bjrcr.20150448 30363635 PMC6180875

